# Health-related quality of life in children with newly diagnosed cancer: a one year follow-up study

**DOI:** 10.1186/1477-7525-4-63

**Published:** 2006-09-20

**Authors:** Markus A Landolt, Margarete Vollrath, Felix K Niggli, Hanspeter E Gnehm, Felix H Sennhauser

**Affiliations:** 1 Department of Psychosomatics and Psychiatry, University Children's Hospital Zurich, Switzerland; 2 Department of Mental Health, Norwegian Institute of Public Health, Oslo, Norway; 3 Institute of Psychology, University of Oslo, Norway; 4 Department of Pediatric Oncology, University Children's Hospital Zurich, Switzerland; 5 Department of Pediatrics, Children's Hospital Aarau, Switzerland; 6 Department of Pediatrics, University Children's Hospital Zurich, Switzerland

## Abstract

**Background:**

Most studies on health-related quality of life (HRQOL) in children with cancer focussed on survivors. Only few studies have evaluated patients during ongoing oncological treatment. The aim of this study was a prospective assessment of HRQOL in children during the first year after diagnosis of cancer and an examination of demographic, medical, and parental predictors of HRQOL.

**Methods:**

Fifty-two patients (mean age: 10.9 years) were assessed 6 weeks and 1 year after diagnosis with the TNO-AZL Questionnaire for Children's Health-Related Quality of Life. Parents completed the Brief Symptom Inventory.

**Results:**

Compared to a community sample, patients reported more physical complaints, reduced motor functioning and autonomy, and impaired positive emotional functioning 6 weeks after diagnosis. HRQOL significantly improved over the year. However, at 1 year, patients still showed reduced motor and emotional functioning. At 6 weeks, children with leukemia were most affected. At 1 year, patients with brain tumors complained about more physical symptoms than the other groups. Intensity of treatment and presence of medical complications mainly influenced HRQOL at 6 weeks but less at 1 year. Parental psychopathology was associated with better cognitive functioning in the child.

**Conclusion:**

This prospective study found several domains of HRQOL to be compromised 6 weeks and 1 year after the diagnosis of cancer. Although HRQOL significantly increased over the year, there were important differences between diagnostic groups. The findings highlight the importance of repeated evaluation of HRQOL in children undergoing cancer treatment and consideration of specific differences between diagnostic groups.

## Background

There has been a dramatic improvement in long-term survival of childhood cancer patients during the past decades. This is related to new approaches to treatment, centralization of care, improved supportive care, and the development of large international clinical trials. Traditionally, progress in pediatric oncology has been measured in terms of survival and rates of response to treatment. Five-year survival rates are now approaching 70% for children with brain tumors and are exceeding 80% for patients with acute lymphoblastic leukemia [1]. However, the same treatments leading to increased survival rates can also cause potentially debilitating physical deficits, such as endocrine dysfunction, neuropsychological deficits or subsequent malignancies. While survival rates and health status are fairly easy to assess, they often do not appropriately mirror the entire impact of the cancer and its treatment on the individual child, particularly with respect to subjective experiences. Such consequences of disease and treatment can be tapped by measures of health-related quality of life (HRQOL). HRQOL is defined as a multidimensional construct composed of the patient's perception of the impact of disease and treatment on his or her functioning in a variety of aspects of life, including the physical, psychological and social domains [2,3]. In children, HRQOL is usually assessed by patients' self report or by parental proxy report.

In the past years there has been an increasing interest in studying HRQOL in pediatric cancer patients. Most studies focussed on survivors and found significant impairment in HRQOL [2,4-6]. Specifically, survivors of CNS tumors seem to be at high risk [5-7]. However, findings are inconsistent and there are also studies that found survivors to have entirely normal HRQOL [8,9]. These inconsistencies can be explained by small sample sizes, wide age range of the children studied, and great variation with regard to type of cancer, treatment, and time elapsed since diagnosis.

Only a few studies have been conducted with pediatric cancer patients during the acute phase of the disease, i.e., during ongoing oncological treatment. Taken together, these studies suggest that HRQOL in children during therapy is significantly lower than in survivors that have completed treatment and in the normal population, respectively [5,9,10]. Also, Magal-Vardi et al. [11] found an association between intensity of treatment and child-reported HRQOL: high-risk children reported poorer quality of life than children with a low or moderate risk. In a recent study, Eiser et al. [12] assessed maternal reports of HRQOL of children with acute lymphoblastic leukemia at 3–6 months and at one year after diagnosis. The authors found a significant improvement of HRQOL scores during the first year after diagnosis. However, some limitations of these studies in acutely ill children merit note. First, medical and psychosocial predictors of HRQOL in acutely ill children have not been studied in detail in a prospective design. Notably, Magal-Vardi et al. [11] used a prospective design, but their sample of 20 children was to small to systematically analyze predictors of HRQOL. Second, most studies of acutely ill children used proxy reports of the child's HRQOL and did not assess the subjective perspective of the individual child [5,10,12].

The aim of the present study was a comprehensive standardized evaluation of self-reported HRQOL in children and adolescents during the first year after a new diagnosis of cancer and a comparison to healthy children. Based on the findings cited above, we hypothesized that children and adolescents with cancer would show a reduced HRQOL during the most acute phase of treatment with improvement over the first year of treatment. Furthermore, we aimed at examining the extent to which sociodemographic, illness-, treatment- and parental characteristics were associated with HRQOL. Children with a higher intensity of medical treatment and more medical complications and children with maladjusted parents were expected to have a reduced HRQOL.

## Methods

### Sample and procedure

The study was approved by the Institutional Review Boards of all study sites. Patients were consecutively assessed over a period of 36 months at four children's hospitals in the German speaking part of Switzerland. The patients and their parents were approached about two weeks after diagnosis if the following criteria were met: 1) new diagnosis of cancer; 2) age between 6.5 and 15 years; 3) no major illness other than cancer; 4) sufficient command of the German language; 5) no evidence of prior mental retardation (physician's rating). Families received written and verbal information about the study and were requested to give signed consent.

Of 83 patients who met these criteria, 22 (10 girls, 12 boys; mean age = 10.43 years) did not participate, mainly because the study seemed too time consuming or because the family felt overwhelmed, leaving 61 patients. Due to incomplete data at either of the two assessments, 9 children (of which three died between the assessments) were excluded from further analyses. The final sample comprised 52 children (response rate 63%). Response rate in parents of the selected children was lower, with 48 mothers and 41 fathers filling in the questionnaires. There were no significant differences between study completers and those who did not participate or had incomplete data with regard to age (t = -1.03; df = 81; p = .31), sex (χ^2 ^= 0.10; df = 1; p = .76), and medical diagnosis (χ^2 ^= 3.40; df = 3; p = .33) of the child. Non-participants had the following diagnoses: leukemia 51.9%, lymphoma 36.5%, brain tumors 13.5%, other solid tumors 17.3%.

Assessments were carried out 6 weeks (T1) and 1 year (T2) after diagnosis. The children were assessed by means of a standardised face-to-face interview lasting from 30 to 45 minutes conducted by trained graduate students of psychology. To ensure that children could express their own feelings and opinions openly, they were interviewed if possible without their parents being present. Parents received their questionnaires at the same time and were asked to complete them separately. Information on demographic and basic medical variables was retrieved from the patients' hospital records. Data on course of treatment and functional status of the child were assessed by questionnaire from the pediatric oncologist in charge of treatment.

## Measures

### Health-related quality of life

Child HRQOL was assessed with the authorized German version of a short form of the TNO-AZL Children's Quality of Life (TACQOL) questionnaire [13]. The TACQOL is a generic instrument designed for HRQOL assessment in medical research and clinical trials. It contains five health status scales: physical functioning, basic motor functioning, autonomy, cognitive and social functioning. In addition, two scales assess general mood: Positive emotional functioning and negative emotional functioning. Following the TACQOL protocol, children were first asked whether a specific problem or symptom had occurred during the two weeks prior to the interview. If affirmed, the child was requested to rate his or her emotional response to the problem on a 4-point Likert-scale. Higher scores represent a better HRQOL. The short form included the original seven scales, but used only four items per scale instead of eight for all scales except one (physical functioning). The short form of the TACQOL was provided from the original authors by using data from the Dutch reference study [13]. By means of a series of reliability analyses the original authors determined which items could be removed from each separate scale with a minimum of loss of scale homogeneity. Following this, a principal component analysis was performed to assess the scale structure of the remaining items. The results supported an internal and external validity of the short form that is comparable to the original TACQOL. Cronbach's alphas of the subscales in the reference sample were between .66 and .77. Norms for this study are provided from a community sample of 1'048 Dutch children [13].

### Parental psychological adjustment

Six weeks after the child's diagnosis mothers and fathers were assessed with the Brief Symptom Inventory (BSI), a 53-item self-report measure assessing the presence and intensity of psychopathological symptoms in adults [14]. The German version of the BSI has been shown to have good reliability and validity [15]. In this study the Global Severity Index (GSI) was used as an overall measure of psychopathology.

### Intensity of oncological treatment

The responsible pediatric oncologist rated treatment intensity on a 3-point scale, with 1 = low: surgery only or six months chemotherapy only or both, with a favorable prognosis (e.g., Hodgkin disease); 2 = medium: treatment longer than 6 months according to the treatment protocol, with an intermediate prognosis (e.g., osteosarcoma); 3 = high: treatment according to high risk protocols, bone marrow transplantation, with a infavorable prognosis (e.g., high-risk leukemia). A very similar scale has successfully been used in a previous study by Magal-Vardi et al. (11).

### Medical complications

The pediatric oncologist in charge was asked to rate medical complications in each patient at 6 weeks and 1 year using a 3-point Likert severity scale: 0 = no complications; 1 = moderate complications (e.g., hospitalization due to infection), 2 = severe complications (e.g., multiple hospitalizations due to infections, no response to treatment).

### Child functional status

Information on functional status with regard to physical activities of daily life was obtained from the pediatric oncologists. They were asked to rate the functional status of patients at 6 weeks and 1 year using a 3-point Likert severity scale: 0 = good functional status, 1 = moderate functional status, 2 = poor functional status. In order to increase interrater reliability, the different degrees of functional impairment were defined in the questionnaire. This measure has successfully been used in an earlier study in children with a chronic disease [16].

### Socioeconomic status

Socioeconomic status (SES) was calculated by means of a score reflecting paternal occupation and maternal education (range 2–12 points). Three social classes were defined as follows: scores 2–5, lower class; scores 6–8, middle class; scores 9–12, upper class. This measure has been shown to be a reliable and valid indicator of SES in our community [17].

### Statistical analyses

Descriptive statistics (means, SD, and frequencies) were calculated for all variables. Differences between the patients and the reference group on HRQOL measures were examined using one-sample Student's *t*-tests. Otherwise, non-parametric statistical techniques were used where possible (Wilcoxon tests for pair-wise comparisons over time, Kruskal-Wallis tests for multiple group comparisons, Spearman-Brown rank correlations to calculate associations), because most of the TACQOL scales showed non-normal distributions. All analyses were performed with two-sided tests and a value of p < .05 was considered significant. Analyses were conducted using SPSS version 10 for Macintosh (SPSS Inc., Chicago, IL, USA).

## Results

### Sample characteristics

Descriptive information about the sample is contained in Table 1. Sex of patients was not equally distributed, with more boys taking part in the study. This is consistent with findings of several international cancer registries [18]. Most families were from the upper- or middle-class, probably due to the language requirement (many non-Swiss nationals are from the lower class). A majority of children suffered from leukemia or malignant lymphoma. However, about 30 percent of the patients were diagnosed with a malignant brain tumor or another malignant solid tumor. The distribution of cancer diagnoses in this sample is similar to that of all newly diagnosed patients in Switzerland aged 6–15 years [18]. Treatment intensity was medium to high in over 80% of the sample. One year after diagnosis, fifty children (96%) had been treated with chemotherapy, 16 (31%) had had surgical interventions, and 18 (35%) had received a radiation therapy. Two children had been treated with a bone-marrow transplantation. One year after diagnosis two children (4%) had had a relapse of the cancer. The average cumulative length of hospital stay was 15.7 days at T1, and 50.2 days at T2. As Table 1 shows, about 56% of the patients had experienced some medical complications in the first 6 weeks after diagnosis. At 1 year the rate of complications was almost 70%. Functional status was rated as being good in only six children (12%) after six weeks, but in 32 children (62%) after one year.

**Table 1:** Demographic and medical characteristics of the sample (n = 52)

**Age at diagnosis (years)**		
Mean (SD)	10.93	(2.62)
Median	10.63	
**Sex**		
Female	20	(38.5%)
Male	32	(61.5%)
**Socioeconomic Status**		
Lower	3	(5.8%)
Middle	38	(73.1%)
Upper	9	(17.3%)
Unknown	2	(3.8%)
**Living with both biological parents**	37	(71.2%)
**Medical diagnosis**		
Leukemia	17	(32.7%)
Malignant lymphoma	19	(36.5%)
Malignant brain tumor	7	(13.5%)
Other malignant solid tumor	9	(17.3%)
**Intensity of therapy**		
Low	9	(17.3%)
Medium	35	(67.3%)
High	8	(15.4%)
**Medical complications at T1**		
None	23	(44.2%)
Moderate	26	(50.0%)
Severe	3	(5.8%)
**Medical complications at T2**		
None	16	(32.7%)
Moderate	31	(59.6%)
Severe	4	(7.7%)
**Functional status at T1**		
Good	6	(11.5%)
Moderate	39	(75%)
Poor	7	(13.5%)
**Functional status at T2**		
Good	32	(61.5%)
Moderate	14	(26.9%)
Poor	4	(7.7%)
Unknown	2	(3.9%)

### Quality of life at 6 weeks and 1 year

Table 2 shows the mean TACQOL scores at 6 weeks and one year for the entire group and broken down by diagnoses, as well as the means of a community sample. Comparisons with the reference group reveal that the entire sample reported a significantly lower HRQOL in several domains. Specifically, at 6 weeks, the patients reported more physical complaints, reduced basic motor functioning and autonomy, and impaired global positive emotional functioning. At one year, motor functioning and positive emotional functioning were still significantly reduced. However, compared to norms, patients now had significantly less physical complaints. Notably, at both assessments, the patients' cognitive and social functioning was normal. Also, the level of negative emotions was not increased in patients.

**Table 2:** Means (SD's) of TACQOL scores at 6 weeks and at 1 year by diagnostic groups

	Norms	All (n = 52)	Leukemia (n = 17)	Lymphoma (n = 19)	Brain tumors (n = 7)	Other solid tumors (n = 9)	p^k^
**TACQOL T1**							
Physical functions	25.3** (5.0)	23.2 (5.3)	20.3 (5.7)	24.4 (4.2)	26.9 (5.0)	22.8 (4.7)	.03
Motor functions	14.8*** (2.0)	11.3 (4.3)	8.8 (4.9)	12.3 (4.1)	13.1 (2.9)	12.3 (2.7)	.06
Autonomy	15.8*** (0.9)	15.0 (1.5)	14.7 (1.8)	15.4 (1.0)	14.7 (1.1)	14.8 (2.1)	.51
Cognitive functions	13.8 (2.6)	14.4 (2.6)	13.6 (3.8)	14.8 (1.5)	15.2 (1.3)	14.3 (2.1)	.70
Social functions	14.4 (1.9)	14.4 (2.2)	13.1 (3.3)	14.8 (1.3)	15.3 (0.5)	15.0 (1.0)	.35
Positive emotions	7.2*** (1.3)	5.9 (1.9)	5.6 (1.7)	5.8 (2.2)	6.0 (2.0)	6.3 (1.7)	.78
Negative emotions	5.6 (1.5)	5.8 (1.7)	5.8 (2.2)	6.0 (1.4)	6.4 (1.3)	4.7 (1.2)	.09
**TACQOL T2**							
Physical functions	25.3*** (5.0)	27.4^††† ^(3.8)	27.1^†† ^(3.7)	28.0^†† ^(3.1)	24.3 (4.6)	29.1^† ^(3.8)	.08
Motor functions	14.8** (2.0)	13.2^† ^(3.6)	13.1^† ^(4.1)	14.4 (1.9)	12.3 (3.5)	11.3 (5.1)	.20
Autonomy	15.8 (0.9)	15.7^†† ^(1.0)	15.4 (1.5)	16.0^† ^(0.0)	15.4 (1.5)	16.0 (0.0)	.17
Cognitive functions	13.8 (2.6)	13.6 (2.5)	13.6 (2.8)	13.5 (2.7)	13.9 (2.5)	13.8 (1.9)	.99
Social functions	14.4 (1.9)	14.7 (1.5)	14.7^† ^(1.8)	14.8 (1.3)	14.0 (1.5)	14.8 (1.3)	.55
Positive emotions	7.2** (1.3)	6.6^† ^(1.5)	6.8^† ^(1.6)	6.8 (1.4)	5.4 (1.4)	6.7 (1.5)	.20
Negative emotions	5.6 (1.5)	5.5 (1.6)	5.7 (1.8)	5.8 (1.6)	5.0^† ^(2.1)	4.9 (0.8)	.57

Note: Significant differences between sample und healthy reference group: ** p < .01; *** p < .001; Significant differences between T1 and T2: ^† ^p < .05; ^†† ^p < .01; ^††† ^p < .001; ^k ^Kruskal-Wallis tests, multiple group comparisons

Comparisons between diagnostic groups with regard to HRQOL revealed that children with leukemia reported significantly more physical symptoms at 6 weeks than children with brain tumors. Motor functioning was also more impaired in patients with leukemia. However, due to the small sample size this difference did not reach statistical significance. At one year, comparisons of diagnostic groups revealed no significant differences. Yet, there was an interesting tendency showing that patients with brain tumors now complained about more physical symptoms than the other diagnostic groups.

Table 2 also shows positive changes in HRQOL in the entire sample between 6 weeks and one year after diagnosis with regard to physical symptoms, motor functioning, autonomy, and positive emotional functioning. Interestingly, cognitive and social functioning and the presence of negative emotions did not change over the course of the disease. These three scales were well within normal ranges at both assessments. Comparisons of subgroups reveal that significant positive changes in HRQOL over time mainly occur in the leukemia and lymphoma groups whereas the children with brain tumors showed a decrease of HRQOL scores.

### Correlates of quality of life

Table 3 reveals associations between individual characteristics of the patients, medical variables, and TACQOL scales at 6 weeks and one year. At both time points, socioeconomic status was not associated with any dimension of HRQOL. However, two demographic variables proved to be important. The age of the child was positively related to autonomy and social functions at 6 weeks and to lower positive emotional functioning at one year. Also, the sex of the child predicted several dimensions of HRQOL at 6 weeks after diagnosis. Girls reported a significantly better functioning in autonomy whereas boys had a better quality of life with regard to cognitive and emotional domains. Interestingly, treatment-related variables, such as intensity of therapy and presence of medical complications mainly influenced HRQOL at 6 weeks but not at one year. Specifically, a more intense and complicated treatment was associated with more physical complaints, more problems in motor functions and a reduced emotional functioning. One year after diagnosis, treatment intensity was less important for HRQOL. However, a significant association between the one-year complication rate and emotional functioning could be found. Functional status at 6 weeks was related to motor functioning at 6 weeks but to none of the HRQOL dimensions at one year. A better functional status at 12 months was associated with better motor and emotional functioning. Finally, Table 3 also reveals significant correlations between parental psychological adjustment at 6 weeks and some of the TACQOL scales. Children reported significantly less cognitive problems if their mothers or fathers had higher scores in the BSI. In addition, paternal psychopathology at 6 weeks was predictive of higher HRQOL with regard to social functioning in the child. Although the correlations are statistically not significant, there is a clear tendency for a negative association of maternal maladjustment with the emotional domains of HRQOL in the acute phase of treatment.

**Table 3:** Spearman correlation coefficients between TACQOL scores and sociodemographic, illness-related and parental variables

	Age	Sex	SES	Intensity of therapy	MC T1	MC T2	FS T1	FS T2	BSI mother T1	BSI father T1
**TACQOL T1**										
Physical functions	-.12	-.06	-.26	-.30*	-.19	-	-.24	-	-.15	-.07
Motor functions	-.07	-.01	-.08	-.34**	-.28*	-	-.49***	-	-.16	-.08
Autonomy	.36**	.28*	.16	.12	-.12	-	-.20	-	-.01	.01
Cognitive functions	-.21	-.33*	-.14	-.03	.19	-	-.05	-	.34*	.32*
Social functions	.31*	-.11	-.16	-.25	.04	-	-.13	-	.10	-.01
Positive emotions	.06	-.02	.17	-.32*	-.36**	-	-.26	-	-.26	.14
Negative emotions	-.17	-.36**	-.26	-.13	-.01	-	-.19	-	-.23	-.08
**TACQOL T2**										
Physical functions	.07	.18	.10	-.01	-.05	-.04	-.14	-.14	.12	.12
Motor functions	.01	.13	.17	.05	.10	-.23	-.12	-.36**	-.11	.04
Autonomy	.24	.08	-.13	.00	-.09	-.24	.01	-.11	.02	-.04
Cognitive functions	-.08	-.06	.00	.03	.13	-.13	-.11	-.09	-.02	-.18
Social functions	.24	-.08	.06	.17	.20	.02	.18	-.08	.27	.31*
Positive emotions	.18	.22	-.07	.11	-.08	-.11	.04	-.24	.06	.23
Negative emotions	.42**	-.04	-.17	-.20	-.10	-.29*	-.11	-.30*	-.05	.14

Note: Medical Complications = MC; Functional Status = FS; * p < .05; ** p < .01; *** p < .001

## Discussion

This prospective one year follow-up study in children and adolescents with newly diagnosed cancer found several domains of HRQOL to be markedly compromised. Notably, compared to a community sample, children reported a diminished quality of life in the physical, motor and emotional domains. In addition, 6 weeks after diagnosis children reported impaired autonomy. The diminishment of quality of life was more pronounced 6 weeks after diagnosis than at the one year follow-up where HRQOL was found to be reduced in only two domains. These results are consistent with our hypothesis and confirm earlier findings by Eiser et al. [10] on significant improvement of mother-reported HRQOL in children with leukemia between a 3–6 months and a one year follow-up. Comparisons between children with different types of cancer in our study revealed that at 6 weeks after diagnosis children with leukemia were the most affected in the majority of dimensions of HRQOL. At one year, however, children with brain tumors seemed to be the most affected although the differences between groups were not statistically significant. This is in line with earlier findings by Meeske et al. [5] who found patients with brain tumors to exhibit more problems than patients with leukemia in the physical, social, psychosocial, and cognitive domains of HRQOL. Clearly, the various groups of pediatric cancer patients are differently affected with regard to their HRQOL. Probably, these differences are due to different treatment protocols. Typically, children with leukemia undergoing chemotherapy suffer from significant treatment-related acute toxicity during the initial induction phase of their treatment protocol. This particular toxicity is less pronounced for children undergoing treatment protocols for brain tumors. This is the first prospective study to show that the most affected diagnostic groups may change over time. Contrary to the suggestion of Meeske et al. [5] we cannot support the notion that patients with leukemia only have minimal changes in HRQOL during the active treatment. In fact, children with leukemia had the most significant improvements between 6 weeks and one year.

This study also analyzed various individual, medical, and parental correlates of child HRQOL. Consistent with our hypothesis medical and treatment variables, such as intensity of therapy and medical complications were associated with HRQOL. As can be expected, the influence of medical variables was more pronounced at 6 weeks than at one year after diagnosis. Thus, the influence of medical and treatment variables on HRQOL gets smaller over the course of treatment which may be due to adjustment processes and a reduction of treatment intensity. However, ongoing medical complications and an impaired functional status still affected emotional domains of HRQOL in a negative way after one year. Thus, increased long-term medical problems negatively impact on emotional functioning. Demographic factors such as age and sex showed particularly strong associations with some domains of HRQOL at 6 weeks but less at one year. In our sample, younger children had a higher risk for HRQOL problems than older children. Also, boys reported fewer problems in the domains of cognitive and emotional functioning. The higher vulnerability of girls with regard to HRQOL problems is well known from other studies [2,5].

To our knowledge this is the first study to show associations between parental psychological adjustment and child self-reported HRQOL in pediatric cancer patients. These findings are all the more important as different informants were used for the assessment of parental mental and child HRQOL, respectively, excluding shared method variance as an explanation. In general, better parental adjustment was associated with better HRQOL in the child, particularly in the emotional domain, six weeks after diagnosis. Surprisingly, however, better maternal and paternal adjustment were associated with poorer HRQOL in the child in the cognitive and social domains. This contradicts previous studies that found family problems to negatively affect HRQOL in children with chronic conditions, such as phenylketonuria or nephrotic syndrome [17,19]. Certainly, the pathways of parental influence on the HRQOL of children with chronic health conditions are not yet well understood and need to be further studied in future.

Strengths of this study comprise its use of multiple sources of information (patients, mothers, fathers, physicians) and its multidimensional and highly standardized assessment of HRQOL in a prospective design. Moreover, sample patients are representative for newly diagnosed children aged 6–15 years in Switzerland. Nonetheless, some limitations merit note. First, our sample is rather small, making statistically significant findings more difficult to achieve and limiting comparisons of diagnostic subgroups. Second, our response rate was only 63%. Although we compared non-participants and participants with regard to demographic characteristics and medical diagnosis we do not know whether these two groups systematically differed regarding their HRQOL. Third, our HRQOL instrument is a generic measure not specifically designed for pediatric cancer. Therefore, it may lack sensitivity for specific problems of this group. However, the TACQOL has successfully been used in a variety of different chronic diseases and has been shown to be a valid and reliable measure allowing comparison with healthy references. Moreover, a German cancer specific HRQOL measure was only published after this study had been completed [20]. Fourth, appropriateness of Dutch HRQOL norms for our sample of Swiss children can be questioned. However, since the Netherlands and Switzerland are European countries with similar social structures, a major cross-cultural bias seems unlikely. This is confirmed by a recent European study in children with chronic diseases that found HRQOL to be higher in Nordic countries compared to Greece and the UK [21]. However, children from central European countries such as Germany and The Netherlands reported very similar HRQOL. This supports the notion that Dutch norms may be used for Swiss children. Finally, there may be some concerns regarding our correlational findings, since the chance of falsely significant results increases with the number of comparisons performed on the same set of data. Because this study had an exploratory character and the limited sample size did not allow multivariate analyses, subsequent studies are needed to confirm the findings.

Our data suggest some possible issues for future research activities. First, this study confirmed that the assessment of HRQOL in children is important and yields valid results. Hopefully, in the future, HRQOL will be considered as an important variable in the evaluation of new medical treatments and standardized HRQOL assessment will be routinely incorporated into therapeutic cancer trials. HRQOL research can be used to optimize treatment and to give important information for decision making if treatment strategies with similar survival rates are compared [22]. Certainly, prospective studies of larger samples of children undergoing active cancer treatment are necessary. Repeated assessments will allow analysis of the course of the disease and the medical, individual and familial predictors of HRQOL over time, as well as more detailed comparisons of different oncological groups. Also, the importance of parental and familial variables on child HRQOL should be studied in more details because this may be important for designing appropriate family-based interventions in children with newly diagnosed cancer. Probably, findings from studies in pediatric cancer patients showing the importance of parental variables for psychological adjustment can be adopted into the research on HRQOL.

There are some clinical implications that can be drawn from this study. Our findings confirm the need for repeated careful evaluation of HRQOL in children who are undergoing active cancer treatment. Our data show that there might be significant differences in HRQOL between diagnostic groups that need to be considered in psychosocial treatment programs in order to improve HRQOL in children with cancer. Psychosocial interventions may not only have to be specifically tailored for diagnostic groups but also for different stages of treatment. Moreover, this study suggests that the whole family needs to be targeted in order to improve the HRQOL in children and adolescents with cancer.

## Competing interests

The author(s) declare that they have no competing interests.

## Authors' contributions

MAL designed the study, participated in the collection of data, analyzed the data and drafted the manuscript. MV designed the study, participated in the interpretation of data and helped to draft the manuscript. FKN helped to design the study and participated in the acquisition and interpretation of data. HEG and FHS participated in the design of the study, the acquisition and interpretation of data. All authors read and approved the final manuscript.
